# Case report: Immunotherapy plus chemotherapy and stereotactic ablative radiotherapy (ICSABR): a novel treatment combination for Epstein-Barr virus-associated lymphoepithelioma-like intrahepatic cholangiocarcinoma

**DOI:** 10.3389/fphar.2023.1147449

**Published:** 2023-08-08

**Authors:** Ruizhen Li, Ke Cheng, Xiaofen Li, Chen Chang, Wanrui Lv, Li Xiaoying, Pei Zhang, Heqi Yang, Dan Cao

**Affiliations:** ^1^ West China Hospital, Sichuan University, Chengdu, Sichuan, China; ^2^ Division of Medical Oncology, State Key Laboratory of Biotherapy, Abdominal Oncology Ward, Cancer Center, West China Hospital, Sichuan University, Chengdu, Sichuan, China

**Keywords:** Epstein-Barr virus-associated lymphoepithelioma-like intrahepatic cholangiocarcinoma, tumor immune microenvironment, immunotherapy, stereotactic ablative radiotherapy, long survival

## Abstract

Epstein-Barr virus-associated lymphoepithelioma-like intrahepatic cholangiocarcinoma (EBVa LEL-ICC) is a rare tumor, characterized by a rich tumor immune microenvironment (TIME). While this tumor is reportedly sensitive to immunotherapy, its response has been inconsistent. This decreased sensitivity was associated with reduced TIME abundance. We report the case of a 53-year-old woman with EBVa LEL-ICC having reduced TIME abundance. The patient presented with a liver lesion, which was detected using ultrasound. Initially, the tumor was sensitive to immunotherapy and chemotherapy (IC), but resistance developed after a short interval. Subsequently, stereotactic ablative radiotherapy (SABR) was added to the patient’s treatment, which now consisted of ICSABR. Successful tumor shrinkage was achieved with the combination therapy regimen. Thus, surgery and ICSABR are effective adjuncts to the first-line IC therapy in improving the survival rate of patients with EBVa LEL-ICC. The results of this study support multidisciplinary treatment as a viable treatment strategy for EBVa LEL-ICC.

## Introduction

Lymphoepithelioma-like intrahepatic cholangiocarcinoma (LEL-ICC) is a rare tumor; it is histologically characterized by dense lymphoid infiltrates interspersed with undifferentiated epithelial cells. LEL-ICC is typically associated with the Epstein-Barr virus (EBV) infection. Hence, it is referred to as EBV-associated LEL-ICC (EBVa LEL-ICC) (Hsu et al., 1996). However, due to its low incidence rate, there is a lack of evidence about the clinicopathological characteristics and standard treatment of LEL-ICC. In previous reports, by analyzing the expression of PD-L1 in LEL-ICC and ordinary intrahepatic cholangiocarcinoma (ICC), it was found that the level of PD-L1 in LELCC was higher than that in ICC, which may indicate the sensitivity to immunotherapy.

EBV-associated cancer is significantly responsive to immunotherapy. This response has been documented in previous cases of stomach, lung, liver, and bile duct cancer (Jiang et al., 2015; Xie et al., 2020; Huang et al., 2021; Lv et al., 2022; Zhu et al., 2022). Its sensitivity to immunotherapy was related to the upregulated expression of programmed death ligand 1 (PD-L1) (Wang et al., 2022). Moreover, the efficacy of immunotherapy and chemotherapy (IC) for advanced cholangiocarcinoma with abundant CD8^+^ T cell infiltration has also been reported; based on this, the tumor immune microenvironment (TIME) was identified as a predictive biomarker of effective immunotherapy (Zhao et al., 2021). However, case reports on the application of immunotherapy for EBVa LEL-ICC treatment are scarce. This study reports the case of EBVa LEL-ICC with reduced TIME abundance. In this study, the 53-year-old woman who presented with a liver lesion was administered with multidisciplinary treatment (MDT), which had long-term efficacy. Thus, MDT is a viable option for rare and refractory cases.

## Case presentation

**FIGURE 1:**
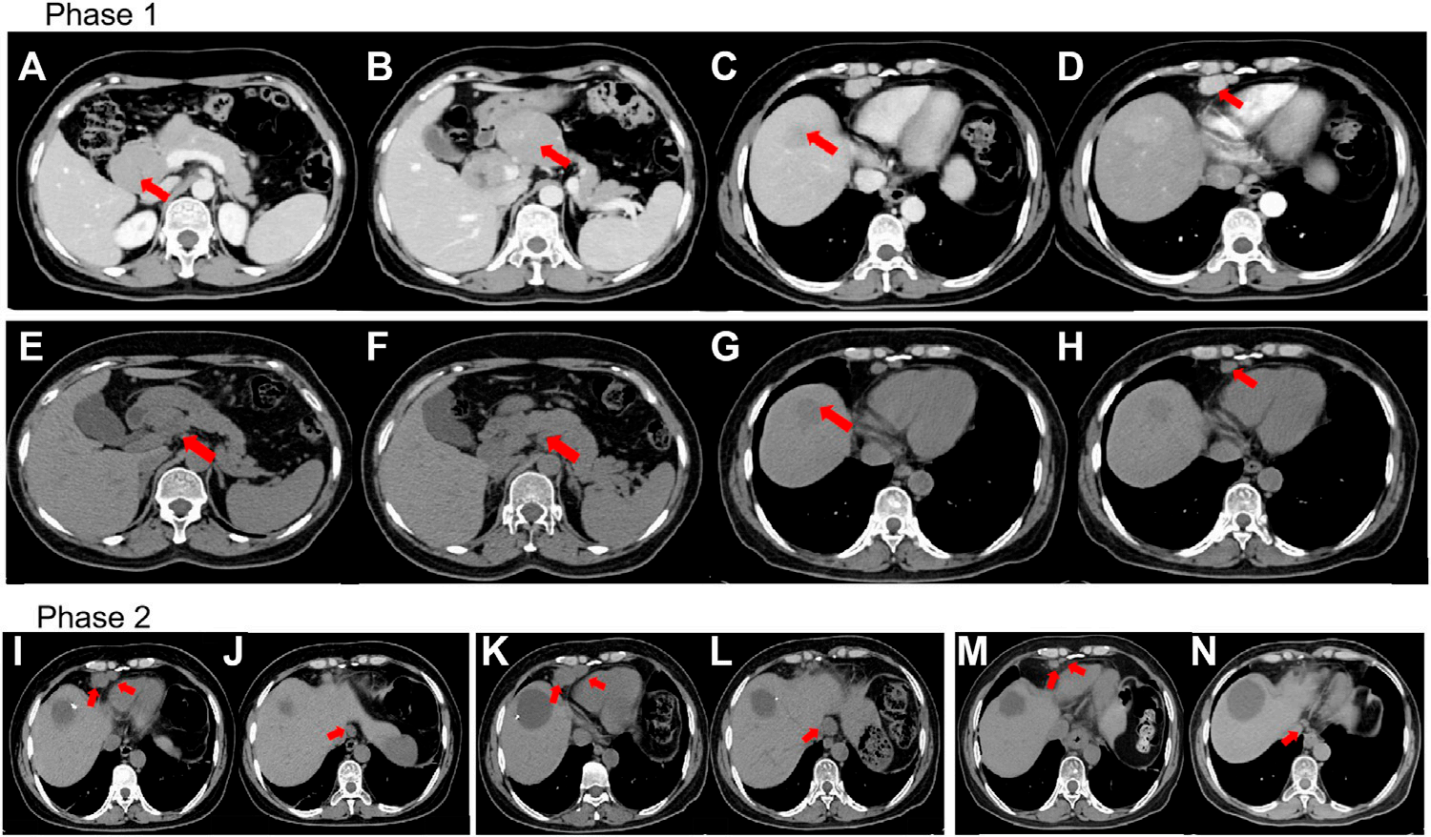
**(A–D)** Prior to treatment, computed tomography (CT) revealed lesions around the pancreas, liver, and lymph nodes located in the cardiophrenic angle. **(E–H)** CT revealed that lesions around the pancreas and lymph nodes reduced in size whereas the lesions proximal to the liver remained constant despite four cycles of immunotherapy and chemotherapy (IC). **(I,J)** CT revealed lymph nodes present even after mesohepatectomy and regional lymph node resection. **(K,L)** CT showed further enlargement of the lymph nodes in the cardiophrenic angle and paraesophageal region despite two cycles of IC. **(M,N)** CT revealed shrinkage of lymph nodes after stereotactic ablative radiotherapy. **(A,B)** and **(E,F)** The red arrow indicates lesions around the pancreas. **(C,G)** The red arrow indicates the lesion in the liver. **(D,H)** The red arrow indicates the lymph nodes in the cardiophrenic angle. **(I,K,M)** The red arrow indicates the lymph nodes located in the cardiophrenic angle. **(J,L,N)** The red arrow indicates the paraesophageal lymph nodes.

**FIGURE 2:**
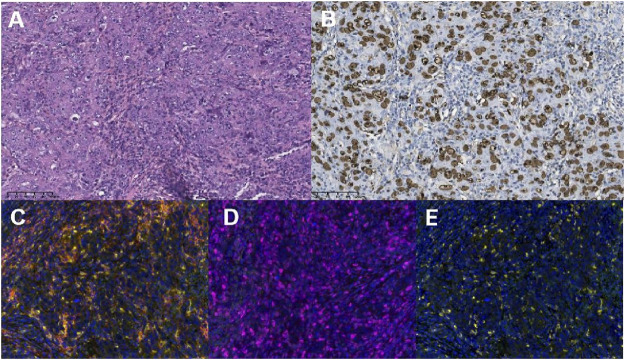
The diagnosis of lymphoepithelioma-like intrahepatic cholangiocarcinoma was pathologically verified with the expression of programmed death-ligand 1 (PD-L1), PD-1, and CD8^+^T cells (×200 magnification). **(A)** Hematoxylin and eosin staining (×200) revealed columnar tumor cells with atypical nuclei that proliferated in a cord-like or glandular tubular pattern. The tumor cells were surrounded by collagen fibers, dense lymphocytic infiltration, and lymphoid follicles. **(B)** The brown cells in EBER-ISH×200 magnification images are the cells harboring Epstein–Barr virus (EBV) infection (EBV-encoded small RNA *in situ* hybridization, EBER-ISH). Representative examples showing immunohistochemical staining of samples that are PD-L1-positive in tumor cells (PD-1^+^ TCs, **(C)**, PD-1-positive in tumor cells [PD-1^+^ TCs, **(D)**], and positive for CD8^+^T cells [CD8^+^T, **(E)**].

A 53-year-old woman presented with a liver lesion, which was detected using ultrasound. The patient had an elevated cytokeratin 19 fragment level (normal range, 30.40 U/mL), but the carcinoembryonic antigen and alpha-fetoprotein levels were normal. Abdominal contrast-enhanced computed tomography (CT) scan revealed a mass, measuring 3 × 3 cm, located in the right lateral hepatic region. Multiple metastatic lesions were also detected in the lymph nodes located in the cardiophrenic angle, hepatic hilar region, and peripancreatic region (Figures 1A–D). A core needle biopsy of the liver was performed, and the histology was consistent with LEL-ICC (Figure 2A). Immunohistochemistry (IHC) analysis revealed that the tumor was positive for PCK and EMA, but it was negative for CK7, PAX8, P63, CK8/18, hepatocyte, Arg, GPC-3, CD30, GATA-3, ER, TTF-1, ALK-1, CDX2, WT-1, and CD34. The tumor tissues were positive for EBV-encoded RNA *in situ* hybridization (Figure 2B). On next-generation sequencing (NGS), genetic aberrations were not identified due to insufficient tumor tissue. According to the eighth edition of the American Joint Committee on Cancer TNM staging system, the tumor was classified under stage IV (T4N2M1).

**FIGURE 3:**
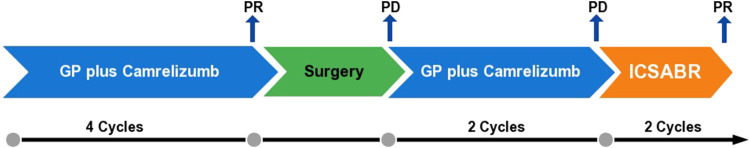
Schematic representation of the anti-tumor therapy process. First-line treatment consisted of gemcitabine (1,000 mg/m^2^, d1, d8, q3w) plus cisplatin (25 mg/m^2^, d1, d8, q3w) and camrelizumab (200 mg, d1, q3w). Subsequently, the surgery was performed. After the surgery, the lymph nodes enlarged; the patient was readministered with GP plus camrelizumab. After two cycles, the tumor further enlarged. The patient was administered with the stereotactic ablative radiotherapy (SABR) combined with camrelizumab and GP (ICSABR), and achieved response after two cycles.

After multidisciplinary consultation, a combination regimen of gemcitabine (1,000 mg/m^2^, d1, d8, q3w) plus cisplatin (25 mg/m^2^, d1, d8, q3w) (GP) and camrelizumab (200 mg, d1, q3w) was administered on June 2021 (Figure 3). After two cycles, the CT scan showed significant regression of the peripancreatic lymph nodes. Based on the Response Evaluation Criteria in Solid Tumors version 1.1 (RECIST 1.1), a partial response was achieved (Figures 1E, F, H). However, a slight enlargement of the liver lesion was noted during the CT scan (Figure 1G). After 4 cycles of IC, the second multidisciplinary consultation had been held, it was concluded that the IC will not lead to regression of the liver lesion; hence, surgery was suggested. On October 2021, a mesohepatectomy and regional lymph node resection were performed. The lymph node in the cardiophrenic angle was difficult to remove due to its proximity to vascular structures (Figures 1I, J). NGS revealed that the liver tissue had a low tumor mutation burden of 3.9 Muts/Mb and a microsatellite stable status. IHC revealed that the tumor tissue had a combined positive score of 20 and a tumor proportion score of 18% (Figures 2C, D). This indicated a high level of PD‐L1 expression in the tumor cells, and the CD8^+^ T cells had infiltrated the tumor cells (Figure 2E). The TIME was examined using multiplex immunohistochemical staining and quantitative analysis (Table 1), but no gene mutations were identified.

**TABLE 1:** Tumor-infiltrating immune cell test results.

Test indicators (multiplex immunohistochemistry)	Test results
CD8^+^T cells	+(2.87%)
PD-L1^+^ cells	+(21.08%)
CD8^+^ PD-1^+^ T cells	+(0.97%)
CD68^+^ macrophage cells	+(13.27%)
CD68^+^ PD-L1^+^ macrophage	+(12.39%)

The postoperative CT scan showed lymph node involvement in the cardiophrenic angle and paraesophageal region (Figures 1I, J). IC was considered effective in the previous treatment. Therefore, administration of IC was continued in the patient. However, the CT showed further 34% enlargement of the lymph nodes after two cycles of IC treatment, which demonstrated progressive disease (PD) (Figures 1K, L). Based on this observation, the patient was diagnosed with progressive disease. Due to the local progression, stereotactic ablative radiotherapy (SABR) for the enlarged lymph nodes with a total dose of 50 Gy was administered in five fractions. Additionally, IC was continued. After two cycles, a partial response was achieved (Figures 1M, N). Currently, PFS reached at least 14 months.

## Discussion

The application of immune checkpoint inhibitors (ICIs) in treating EBVa LEL-ICC has not been discussed in previous studies. The efficacy of immunotherapy has been documented for LEL-ICC of the lungs, breast, bladder, and liver (Iezzoni et al., 1995). Despite the lack of clinical evidence, there are biologic reasons supporting the potential efficacy of ICIs in treating EBVa LEL-ICC. Compared with the conventional cholangiocarcinoma, EBVa LEL-ICC has an increased proportion of intratumoral lymphocytes; this proportion was reportedly a predictor of the response of various cholangiocarcinoma subtypes to ICIs (Huang et al., 2021).

In this case, the patient suffered from unresectable advanced cholangiocarcinoma at the time of diagnosis. However, there was not obvious clinical symptoms or no similar family history. The ABC-02 study, published in 2010, suggested that the administration of GP in patients with locally advanced or metastatic cholangiocarcinoma was associated with a significant survival advantage without the addition of substantial toxicity. The objective response rate for this regimen was 23% (Valle et al., 2010). In the latest National Comprehensive Cancer Network guidelines, GP combined with durvalumab was recommended as the first-line treatment for advanced unresectable cholangiocarcinoma because it significantly prolonged progression-free survival and overall survival (OS) (Oh et al., 2022). A study, published in 2020 and reported by the American Society of Clinical Oncology, suggested that gemcitabine and oxaliplatin combined with camrelizumab achieved a median OS of 11.8 months (95% CI 8.3–15.4) for patients with advanced cholangiocarcinoma (Chen et al., 2020). Based on the current data on IC as the first-line treatment for cholangiocarcinoma, patients with EBVa LEL-ICC are expected to benefit from immunotherapy due to the pathological characteristics of the tumor. In this case, GP and camrelizumab were chosen.

A previous study revealed significant differences in the immune infiltrate level within the microenvironment between tumor metastasis sites (Conway et al., 2022). Metastatic liver lesions had lesser number of intratumoral lymphocytes than distant lymph node metastases, and this difference has a potential effect on immunotherapy outcomes (Conway et al., 2022). In this case, the sizes of the lymph nodes were significantly reduced, but the liver lesion exhibited minimal improvement. This was attributed to the difference in TIME between liver and lymph node metastasis. After consultation of our multidisciplinary doctors, it is thought that liver tumors were not sensitive to the IC, while tumor lesions in lymph nodes responded well. Considered that it was possible to radically treat lymph node lesions through radiotherapy in subsequent treatment, we decided to perform tumor reduction surgery on the liver lesion which also evaluating the tolerance of the patient. The TIME of the liver lesion consisted of CD8^+^, PD-L1^+^, and CD8^+^ PD-1^+^ T cells (2.87%, 21.08%, and 0.97%, respectively).

Postoperatively, enlarged lymph nodes were noted in the cardiophrenic angle and paraesophageal region. As the lymph nodes were sensitive to IC during the previous treatment, the same treatment regimen was reintroduced for two cycles. However, the expected tumor regression was not attained. Compared with a previous case of EBV-associated gastric cancer, the present case had a less abundant TIME, such as CD8^+^ T cell density (10.59% vs. 2.87%) (Lv et al., 2022). Compared with EBVa ICC, EBVa LEL-ICC had significantly increased densities of CD8^+^ T cells (Huang et al., 2021). The retrospective study also suggested that infiltration of the CD8^+^ T cells significantly increased local immune activation, and this was associated with a more favorable prognosis and increased responsiveness to immunotherapy (Song et al., 2010; Huang et al., 2021). Meanwhile, in previous studies, it has been found that tumor TIME is upregulated after chemotherapy, and the induction of innate immune components, including macrophages and NK cells, drives antigen presentation. However, due to the lack of pre treatment samples from patients, it was not confirmed in our study. However, as the lesion in this case had a less abundant TIME, the patient had refractory disease with a complicated course.

Thus, MDT was performed to facilitate a more comprehensive management plan. After undergoing tumor reduction surgery, the patient underwent radiotherapy (RT) and immunotherapy for the lymph nodes. SABR is defined as a radiation dose of more than 5 Gy/fraction with a high compliance and sharp dose drop to protect the surrounding organs at risk. ISABR is a novel therapeutic option involving both SABR and immunotherapy (Weichselbaum et al., 2017). Its effectiveness has been documented in previous studies. The combination of SABR and ipilimumab, an anti-CTLA-4 monoclonal antibody, was reportedly effective in treating metastatic melanoma in 2011 (Hiniker et al., 2012; Grimaldi et al., 2014). A study in 2013 determined the absolute effect of ipilimumab combined with SABR on metastatic liver cancer (Golden et al., 2013). According to several studies, SABR stimulated the systemic immune response, thus leading to enhanced recognition of tumor cells by the immune system and neoplastic cell death (Loblaw et al., 2013; Norkus et al., 2013). In an ongoing trial COSINR, the immune signatures in tumor tissue before and after radiotherapy combined with immunotherapy were analyzed. It was found that SABR combined with ICIs increased the expression of adaptive immune and cytotoxic T cell gene programs, and improved tumor cell elimination over SABR alone. However, the study also demonstrated that SABR alone was insufficient to induce local immune augmentation. In this case, the combination of IC and SABR, ICSABR, successfully elicited a treatment response might support previous studies. This case demonstrated that ICSABR is an effective treatment option for refractory EBVa LEL-ICC. Meanwhile, the liver function and leukocyte in serum were monitored and within the normal range, which also confirmed the safety of the ICSABR. The combination treatment resulted in a marked reduction of tumor size and a more favorable long-term survival.

## Conclusion

In this case, the flexible application of ICSABR in a patient with a rare and refractory EBVa LEL-ICC achieved favorable outcomes. To the best of our knowledge, this is the first report on the systematic comprehensive treatment of EBVa LEL-ICC. However, as our research was a case report, we still need randomized controlled clinical study to further verify its efficacy. With the development of anti-tumor treatment, combining multiple therapeutic options may increase the efficacy of the treatment regimen. This case will serve as a reference for future large-scale prospective clinical studies.

## Data Availability

The raw data supporting the conclusion of this article will be made available by the authors, without undue reservation.
